# Statistical issues in the design of randomised surgical trials: a practical example of the possible solutions

**DOI:** 10.1186/1745-6215-12-S1-A100

**Published:** 2011-12-13

**Authors:** Helen Marshall, Ivana Holloway, Julia M Brown

**Affiliations:** 1 Clinical Trials Research Unit, University of Leeds, Leeds, UK

## Objectives

Surgical randomised trials often have unique design and implementation issues: this abstract presents three key statistical issues accounted and the solutions adopted during the design of an international, randomised, surgical trial (ROLARR), comparing laparoscopic versus robotic-assisted surgery for rectal cancer patients, performed at the Clinical Trials Research Unit.

## Methods and results

One significant issue for surgical trials is to ensure surgeons recruiting to the trial are over their initial ‘learning curve’. To ensure surgeon competency and minimise any ‘learning curve’ effect, ROLARR only includes surgeons who have performed at least 30 rectal cancer resections (minimum of 10 for each procedure). Randomisation also stratifies by surgeon to ensure balance between arms within each surgeon and within the stage that individual surgeons have reached on the learning curve. To be able to statistically assess the learning curve at analysis, data on time-dependent factors known to influence the learning curve will also be collected prior to and on a regular basis during recruitment.

Another issue is blinding. Blinding surgical teams is generally impossible, but blinding patients may be feasible. In ROLARR however, although patients could be initially blinded to their performed surgery, maintaining the blind was felt to be difficult to achieve successfully. ROLARR therefore incorporates objectives measures and central blinded assessments of these measures to reduce potential bias.

Timing of randomisation can also be problematic due to the need for theatre planning. Preferably surgery should take place as soon as possible after randomisation however in ROLARR, up to 28 days after surgery has had to be permitted. Monitoring timings will take place to allow prompt action on any possible problems that may introduce bias.

## Conclusions

Surgical trials are complex to design and implement. Careful consideration needs to be given to the additional issues that arise to ensure an accurate and unbiased interpretation of the results.

